# Oxidative stress biomarkers in pregnancy: a systematic review

**DOI:** 10.1186/s12958-024-01259-x

**Published:** 2024-08-02

**Authors:** Abubakar Ibrahim, Martina Irwan Khoo, Engku Husna Engku Ismail, Nik Hazlina Nik Hussain, Anani Aila Mat Zin, Liza Noordin, Sarimah Abdullah, Zaleha Abdullah Mahdy, Nik Ahmad Zuky Nik Lah

**Affiliations:** 1 Department of Obstetrics and Gynaecology, School of Medical Sciences, Universiti Sains Malaysia, Kubang Kerian Kelantan, 16150 Malaysia; 2 Department of Chemical Pathology, School of Medical Sciences, Universiti Sains Malaysia, Kubang Kerian Kelantan, 16150 Malaysia; 3 Women’s Health Development Unit, School of Medical Sciences, Universiti Sains Malaysia, Kubang Kerian Kelantan, 16150 Malaysia; 4 Department of Pathology, School of Medical Sciences, Universiti Sains Malaysia, Kubang Kerian Kelantan, 16150 Malaysia; 5 Department of Physiology, School of Medical Sciences, Universiti Sains Malaysia, Kubang Kerian Kelantan, 16150 Malaysia; 6 Biostatistics and Research Methodology Unit, School of Medical Sciences, Universiti Sains Malaysia, Kubang Kerian Kelantan, 16150 Malaysia; 7 Department of Obstetrics and Gynaecology, Universiti Kebangsaan Malaysia Medical Centre, Cheras, Kuala Lumpur 56000 Malaysia

**Keywords:** Pregnancy, Oxidative stress, Biomarkers, Antioxidants, Pre-eclampsia, Fetal growth restriction

## Abstract

**Background:**

This systematic review explores the level of oxidative stress (OS) markers during pregnancy and their correlation with complications. Unlike previous studies, it refrains from directly investigating the role of OS but instead synthesises data on the levels of these markers and their implications for various pregnancy-related complications such as preeclampsia, intrauterine growth restrictions, preterm premature rupture of membranes, preterm labour, gestational diabetes mellitus and miscarriages.

**Method:**

**Study Design:**

Utilizing a systematic review approach, we conducted a comprehensive search across databases, including MEDLINE, CINAHL (EBSCOhost), ScienceDirect, Web of Science, and SCOPUS. Our search encompassed all publication years in English.

**Results:**

After evaluating 54,173 records, 45 studies with a low risk of bias were selected for inclusion. This systematic review has underscored the importance of these markers in both physiological and pathological pregnancy states such as preeclampsia, intrauterine growth restrictions, preterm premature rupture of membranes, preterm labour, gestational diabetes mellitus and miscarriages.

**Conclusion:**

This systematic review provides valuable insights into the role of OS in pregnancy and their connection to complications. These selected studies delved deeply into OS markers during pregnancy and their implications for associated complications. The comprehensive findings highlighted the significance of OS markers in both normal and pathological pregnancy conditions, paving the way for further research in this field.

**Supplementary Information:**

The online version contains supplementary material available at 10.1186/s12958-024-01259-x.

## Introduction

Oxıdatıve stress (OS) biomarkers can be categorized into two groups: molecules that undergo modifications due to interactions with reactive oxygen species (ROS) within their surroundings, and molecules within the antioxidant system that alter in response to heightened redox stress [1]. OS markers in pregnancy refer to specific biological indicators or measurements that are used to assess the presence and extent of OS in pregnant individuals [2]. OS occurs when there is an imbalance between the production of reactive oxygen and nitrogen species (ROS and RNS) and the body’s ability to counteract their harmful effects through antioxidant defenses [3].

In pregnancy, the increased metabolic demands and physiological changes can make women more susceptible to OS [4]. These markers are used to evaluate the potential impact of OS on maternal and fetal health and may help in understanding and managing pregnancy complications associated with OS, such as infertility, miscarriage, pre-eclampsia and fetal growth restriction. Researchers and healthcare professionals often use these markers to monitor and study OS during pregnancy [5, 6].

Numerous research studies have documented the presence of OS markers in pregnancy; however, these studies are conflicting. Furthermore, these studies tend to focus on the increase of specific OS markers in limited pregnancy conditions, such as pre-eclampsia [3, 7–19], intrauterine growth restriction [20–26], gestational diabetes mellitus [17, 25, 27–34], premature rupture of membranes [35–43], polycystic ovary syndrome [44], miscarriage [45, 46], and preterm labor [37, 35, 40, 47–49].

Additionally, some studies only address the elevation of these markers during certain trimesters of pregnancy [46, 50–53] or postpartum [6, 54–57]. To address this existing research gap comprehensively, our systematic review aims to provide a comprehensive and detailed analysis of OS markers throughout pregnancy, thus offering a comprehensive overview of the subject.

## Materials and methods

### Study design and search strategy

A systematic review was conducted by synthesizing available studies, aiming to address the prevailing gaps and inconsistencies in the literature regarding OS markers in pregnancy. This comprehensive analysis was designed to shed light on areas requiring further investigation. The review adhered to established guidelines for conducting systematic reviews, including the Preferred Reporting Items for Systematic Reviews and Meta-Analyses (PRISMA) [58] and the Cochrane Handbook for Systematic Reviews of Interventions [59]. By following these rigorous protocols, the study sought to ensure the highest standards of methodological rigor and transparency in the review process, thereby contributing to a more robust understanding of the role of OS markers in pregnancy.

A comprehensive systematic search was undertaken by three independent reviewers across various medical and scientific databases, including MEDLINE, CINAHL (EBSCOhost), ScienceDirect, Web of Science, and SCOPUS, without limitations on publication year. The search strategy involved utilizing a combination of search terms and MeSH terms to comprehensively cover the topic of OS biomarkers in pregnancy. The combined terms used are: ((Pregnancy OR gestation OR “pregnant toxemia” OR obstetrics OR gynecology OR “gyn(a)ecology”) AND (“oxidative stress” OR biomarkers OR “total oxidative status” OR “total antioxidant capacity” OR “lipid peroxidation” OR peroxides OR “hydrogen peroxide”) AND (“malondialdehyde” OR malonaldehyde OR “thiobarbituric acid reactive substances” OR “protein carbonyls” OR “nitric oxide” OR “advanced oxidation protein products” OR “advanced glycation end products” OR “carboxymethyl-lysine”)) NOT ANIMALS NOT “genetic studies and several other related terms. The primary objective of this systematic search was to identify research papers that not only presented the parameters used to characterize OS and its markers but also explored the association between OS and pregnancy, along with pregnancy-related complications.

We retrieved all studies published in these databases from their beginning until October 22, 2023, to evaluate their suitability for inclusion in this research. Our search encompassed full-text articles in English language. To uncover more potentially suitable studies, we also examined the reference lists of the included citations. The detailed search terms used are listed in Table 1.

**Table 1:** Search terms for systematic review on OS markers in pregnancy

Category	Search terms
Pregnancy related terms	((Pregnancy OR gestation OR “pregnant toxemia” OR obstetrics OR gynecology OR “gyn(a)ecology”)
Oxıdatıve stress-related terms	(“oxidative stress” OR biomarkers OR “total oxidative status” OR “total antioxidant capacity” OR “lipid peroxidation” OR peroxides OR “hydrogen peroxide”)
Specıfıc oxıdatıve stress markers	(“malondialdehyde” OR malonaldehyde OR “thiobarbituric acid reactive substances” OR “protein carbonyls” OR “nitric oxide” OR “advanced oxidation protein products” OR “advanced glycation end products” OR “carboxymethyl-lysine”))
Exclusıon	NOT ANIMALS, NOT “genetic studies”

### Eligibility criteria

The eligibility criteria for our systematic review were established to ensure a comprehensive and focused analysis of OS markers in pregnancy and pregnancy-related complications. Pregnant women were the target population of interest, and studies investigating OS markers in pregnancy and their association with pregnancy-related complications were included. A comparison between healthy pregnancies and high-risk pregnancies was considered where applicable. The primary outcomes of interest were levels of OS biomarkers, and included studies were required to present measures of central tendency (mean, median) and variability (standard deviation, interquartile range) for these biomarkers. Both interventional and observational studies were included to provide a comprehensive understanding of the topic, and studies conducted at healthcare institutions were included to ensure clinical relevance and applicability. Only full-text articles published in English were considered for inclusion. Animal experiments, studies with incomplete data on OS markers, genetic studies of antioxidant enzymes, and grey literature, including case series/reports, conference papers, proceedings, abstract-only articles, editorial reviews, letters of communication, and commentaries, were excluded to uphold the quality and reliability standards of included studies.

#### Ratıonale for ınclusıon and exclusıon crıterıa

To ensure the comprehensiveness and relevance of our systematic review, we adopted a comprehensive approach in selecting studies related to OS markers in pregnancy and pregnancy-related complications. This approach involved including a diverse range of studies, both interventional and observational, to provide a thorough understanding of OS markers across different study designs and populations. We also considered studies conducted at healthcare institutions to ensure the clinical relevance of our findings.

In our selection process, we specifically excluded animal experiments, studies with incomplete data on OS markers, genetic studies of antioxidant enzymes, and grey literature such as case series/reports, conference papers, proceedings, abstract-only articles, editorial reviews, letters of communication, and commentaries. These exclusion criteria were put in place to maintain the focus on human pregnancy, ensure data completeness and reliability, and uphold the quality and reliability standards of the included studies.

Additionally, we included studies that measured OS biomarkers in various biological fluids (e.g., serum, urine, amniotic fluid) to capture a comprehensive range of data and avoid potential biases. These comprehensive inclusion criteria are essential to ensure that we do not miss relevant studies that could contribute significantly to our understanding of OS in pregnancy and its associated complications.

### Study selection and screening

Our search strategy retrieved all relevant records, which were then imported into Zotero and EndNote software. Duplicate articles were eliminated. Three separate reviewers evaluated the titles and abstracts of the identified articles. For eligible studies, the full-text articles were carefully reviewed to determine their suitability. In cases of disagreement between the three reviewers, a consensus discussion was held, and a fourth reviewer was consulted when necessary.

### Quality assessment and bias

A critical evaluation was conducted to gauge the quality of data in the context of JBI Systematic Reviews, using The Joanna Briggs Institute Critical Appraisal tools [60]. Three independent reviewers assessed biases. They categorized bias risk as low when more than 70% of the responses were affirmative, moderate when 50%-69% were affirmative, and high when up to 49% were affirmative. Studies demonstrating high or moderate bias risk were omitted from the review.

## Results

### Study characteristics

**Fig. 1:**
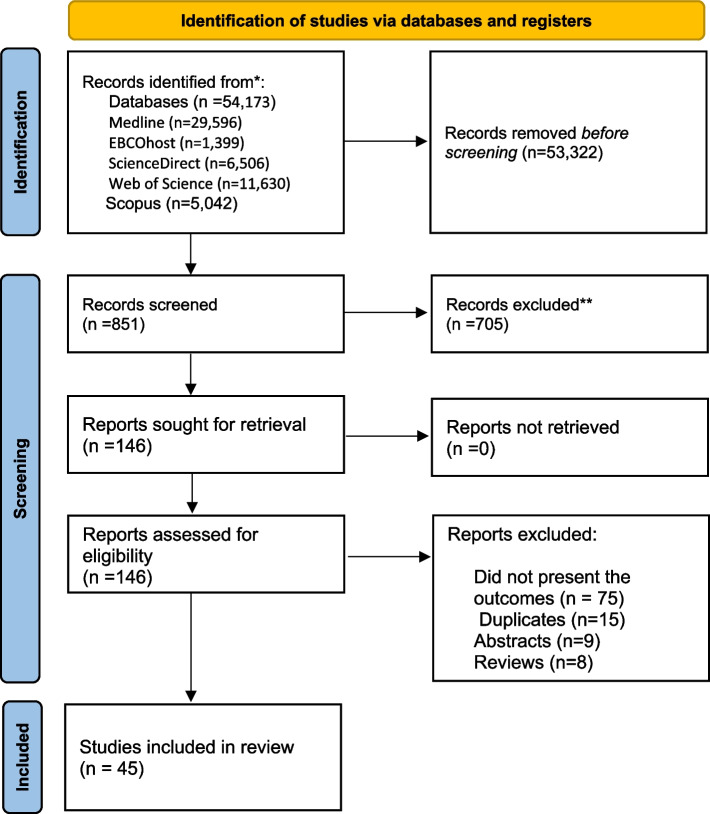
Prepared reporting item for systematic review and meta-analysis. (*n* = number of records)

A comprehensive electronic search using diverse search terms resulted in the retrieval of 54, 173 articles. Following the evaluation of their titles and abstracts, 53, 322 articles were eliminated. Subsequently, 851 articles were assessed for eligibility, with 146 of them undergoing further evaluation. In the end, 45 papers met the criteria and were included in the final review. This screening process for inclusion and eligibility was carried out by three independent reviewers. This systematic review encompassed various study designs, comprising 16 cross-sectional studies, 18 case-control studies and 11 cohort studies. The PRISMA flow chart (Fig. 1) illustrates the search process.

### Oxidative stress markers

In this systematic review, we meticulously examined the landscape of OS markers studied during pregnancy, aiming to unravel the intricate mechanisms underlying these complications.

Throughout the literature, researchers have extensively investigated various OS markers to gauge the physiological state of pregnant individuals. Noteworthy among these markers are molecules such as malondialdehyde (MDA), 8-hydroxydeoxyguanosine (8-OhdG), and thiobarbituric acid reactive substances (TBARS) etc. These markers serve as sentinel indicators, offering unique insights into different facets of OS dynamics during pregnancy.

However, the scope extends beyond these highlighted markers, encompassing a diverse array of molecules and enzymes that collectively contribute to our understanding of OS in pregnancy. Each marker, meticulously studied and analyzed, enriches our comprehension of the underlying mechanisms and pathways implicated in pregnancy-related complications. For a comprehensive overview of the OS markers explored in the literature, please refer to Table 2.

**Table 2:** Detailed descriptions of oxidative stress markers in pregnancy

Marker	Description
Malondialdehyde (MDA)	An indicator of lipid peroxidation [61]
8-hydroxydeoxyguanosine (8-OHdG)	A marker for oxidative DNA damage [50]
Thio barbituric Acid reactive substances (TBARS)	An Indicator of lipid peroxidation products [62]
Nitrous oxide (NO)	Associated with nitrosative stress [63]
Derivatives of reactive oxygen metabolites (dROM)	Measures the levels of reactive oxygen species (ROS) in a biological sample, indicating oxidative stress [64]
Total antioxidant capacity (TAC)	Reflecting the global antioxidant defence [61]
Advanced oxidation protein products (AOPP)	Assessing protein oxidation [65]
Superoxide dismutase (SOD)	An enzyme combating superoxide radicals [66]
Glutathione peroxidase (GPx)	Involved in reducing hydrogen peroxide and lipid peroxides [16]
Glutathione reductase (GR)	Participating in the regeneration of reduced glutathione [67]
Lipid peroxidation (LPO) or (LOOH)	Indicating oxidative damage to lipids [63]
Xanthine oxidase (XO)	An enzyme associated with oxidative stress of purine metabolites [68]
Oxidized glutathione (GSSG)	A measure of the oxidized form of glutathione [69]
Catalase (CAT)	An enzyme breaking down hydrogen peroxide [6]
Paraoxonase (PON-1)	Associated with oxidative stress and inflammation [65]
Oxidative stress index (OSI)	Providing an overall index of oxidative stress [48]
Total free sulfhydryl (-SH)	Measures the concentration of sulfhydryl groups (-SH) in a sample, indicating antioxidant levels [70]
8-iso-prostaglandin F2α (8-iso-PGF2α)	Indicative of oxidative stress effects on prostaglandins [71]
Prostaglandin F2α (PGF2α)	Related to blood pressure regulation and inflammation [42]
Glutathione (GSH)	A critical antioxidant molecule [53]
Glutathione transferase (GST)	An enzyme involved in detoxification processes [72]
Myeloperoxidase (MPO)	Indicative of neutrophil activation and oxidative stress, carbonylated proteins—reflecting protein oxidation [73]
Advanced glycation end products (AGEs)	Associated with glycation-induced oxidative stress [74]

The findings of these studies underscored the relative importance of these markers in both physiological and pathological pregnancy conditions.

In this systematic review, we meticulously dissect the findings into distinct sections, offering a captivating exploration of OS markers. Our examination encompasses both the realm of normal physiological pregnancies and the intricate web of pathological conditions, including pre-eclampsia (PE), intrauterine growth restrictions (IUGR), preterm labor, preterm birth (PTBs), preterm-premature rupture of membrane (pPROM), gestational diabetes mellıtus (GDM), miscarriages. We really hope this systematic review will stand as a beacon of thorough exploration and a testament to the evolving landscape of scientific inquiry.

#### OS markers in physiological pregnancy (normal pregnancy conditions)

We analyzed seven studies that investigated OS markers during physiological pregnancies. Notably, six of these studies diligently controlled factors known to influence OS levels, including vitamin supplementation, age, smoking, alcohol consumption, physical activity, and overall health status. Despite these precautions, there was a discernible elevation in various OS markers.

In the first study, Zelanzniewick et al 2015, which was conducted during the first trimester, measured 8-isoPG F2α and 8-OHdG, revealing a notable increase in these markers [50]. In the second study, [60] monitored MDA and TAC levels throughout pregnancy, MDA exhibited consistent increases in all trimesters, with the highest levels observed in the first trimester. Meanwhile, TAC levels reached their zenith during the third trimester [61].

In the third study, of [72] focusing on the second trimester, measured 8-Ip and 8-OHdG, uncovering an elevation in these markers [75]. In the fourth study, Restini et al. 2018 found out that pregnant women exhibited significantly higher levels of TAC and SOD compared to non-pregnant women, whose values remained within the normal range [66]. In the fifth study, Roger et al. 2007 concentrated on 8-isoPG F2α and 2,3 8-isoPG F2α, unveiling an increase in isoprostane levels among normotensive pregnant women [71]. In the sixth study [61] assessed TBARS levels, revealing a rise in this marker among normotensive pregnant women [62]. Finally, In the seventh study, Vakilan et al. 2009 examined TBARS levels at birth, indicating a significant increase in this marker [76] .

#### OS markers in Preeclampsia (PE)

This systematic review encompasses 11 articles that investigate OS markers in PE. However, akin to most studies, these research endeavors also exhibit certain limitations as they do not comprehensively cover all potential markers. Instead, each study focuses on a specific subset of these markers.

In the first study, Jjain and Wise 1995 centered on MDA and revealed a significant increase in its levels in cases of PE when compared to normal pregnancies [77]. In the second study, [18] examined 8-isoPG F2α and MDA, also uncovering a noteworthy elevation in these markers in contrast to normal pregnancy [18]. In the third study, [11] assessed SOD, highlighting its significant increase in PE compared to healthy pregnancies [11]. In the fourth, study [13] scrutinized TBARS, and its outcomes demonstrated a significant rise in PE cases in comparison to the control group [13].

In the fifth study, [16] ventured into the assessment of SOD, CAT, and GPx, all of which exhibited substantial increases in PE cases as opposed to normal conditions [16]. In the sixth study, Bogavac et al. 2016 delved into the measurement of SOD, GSH-Px1, and TAS, revealing significant elevations in these markers in PE compared to control [53].

In the seventh study Ahmed et al. 2019, focusing on SOD, GSH, and GSG, indicated significant increases in PE cases [69]. In the eighth study, Ferguson et al. 2015 analysed 8-isoprostane, which exhibited a notable increase in PE [78]. In the ninth study, Sikkema et al. 2001 homed in on SOD, showing a significant elevation in PE [8]. In the tenth study, Shigemitsu et al. 2016 investigated MDA and TBARS in the second and third trimesters of PE subjects, with a substantial increase, particularly in the third trimester [79]. And lastly. In the eleventh study, Godhamgaonkar and Joshi 2023 assessed MDA, and the results showed significant increase in the marker in PE patient as compared control group [80].

#### OS markers in Intra Uterine Growth Restriction (IUGR)

In this section of our systematic review, we meticulously analysed nine studies that rigorously adhered to our stringent inclusion criteria. These investigations sought to elucidate the intricate landscape of OS markers within the context of IUGR.

In the first study [68] conducted a comprehensive assessment of TOS, -SH, and TAC. The results unveiled a conspicuous upsurge in these markers [70]. The second study [20] focused on TOC and TAC. Its findings showcase a significant increase in these markers in cases compared to controls [20]. In the third study, [66] assessed MDA, a prominent marker within the OS realm, exhibiting a marked increase in IUGR cases [81]. In the fourth study, [66] measured MDA, CAT, XO, and SOD. Strikingly, these markers demonstrated substantial elevations within the context of IUGR [68].

In the fifth study, [62] focused on 8-OHdG and dROM. Both markers demonstrated significant rises in IUGR compared to normal pregnancy conditions [64]. In the sixth study, [71] assessed F2α-Isoprostavne, unveiled a significant increase in cases compared to controls [82]. In the seventh study, [26] measured MDA, The results emphasize its significant increase in IUGR compared to normal pregnancy conditions [26]. In the eighth study, Zhang et al. 2023 measured 8-OHdG, revealing a significant elevation, particularly in cases of IUGR [52]. Finally, In the ninth study, [21] assessed MDA and XO. Both markers demonstrated significant increases in cases of IUGR [21], underscoring the pervasive influence of oxidative stress in the context of IUGR.

#### OS markers in Preterm Prelabour Rupture of Membrane (pPROM), Preterm Birth (PTBs) and Preterm labor

While these conditions differ in their specific triggers and symptoms, they are all characterized by the shared risk of premature delivery. pPROM can result in PTBs. pPROM, which is defined as the rupture of the amniotic membranes before reaching 37 weeks of gestation, occurs in around 4% of pregnancies. It directly precedes approximately 40% to 50% of all cases of spontaneous PTBs [37]. Which is why we have included them in the same section. Within this section, we produced separate subsections, dedicated to explaining the biomarkers of OS that are related to one of these complications.

### Prelabour Rupture of Membrane (PROM) and pPROM

PROM is defined as the rupture of the amniotic membranes after 37 weeks of gestation. Five studies examining biomarkers of OS in pPROM and PROM were included in this analysis. In the first study, [39] analyzed 8-Isoprostane, TOS, and TAS, revealing an increase in TOS and 8-Isoprostane levels and a decrease in TAS within the case groups in comparison to the control groups [39]. In the second study, [35] evaluated TAC and TBARS, demonstrating a significant increase in these markers in PROM patients compared to normal pregnant women [35]. In the third study, [42] investigated Isoprostane and F2α IP, presenting significant increases in these markers in cases relative to the control group [42].

In the fourth study, Ryu et at. 2017 scrutinized MDA, revealing a significant increase in MDA levels in PROM patients when compared to normal pregnant women [38]. In the fifth study, [43] delved into TAS and TOS in pPROM cases, unveiling significantly higher TOS levels and lower TAS levels in pPROM patients compared to the normal health control group with healthy amniotic membranes [43].

### Preterm birth/Preterm labour

Five studies investigated various OS markers in complications related to PTBs. In the first study, Cindrova davies et al. 2018 examined 8-OHdG and H_2_O_2_, revealing a significant increase in these markers in cases compared to controls [83]. In the second study, [77] analyzed 8-iso-PGF2α in PTB women and normal pregnant women, uncovering a significant increase in this marker in PTBs women [84]. In the third study, Kurlak et al. 2014 measured TBARS, demonstrating a significant increase in these markers in preterm Women compared to the control group [6]. In the fourth study, Venkatesh et al. 2016 assessed 8-OHdG and 8-isoprostane, and the results indicated a significant increase in these markers [85].

In the final study study, Hamzaoglu et al. 2023 measured SOD, TOS, OSI, and TAS. The results indicate an increase in SOD, TOS, and OSI within the case group, along with a decrease in TAS compared to the control group. Notably, TAS exhibited higher levels in the control group in contrast to the case group [48].

#### OS markers in Gestational Diabetes Mellitus (GDM)

This systematic review incorporated four studies that met our eligibility criteria, focusing on OS markers in the context of GDM. In the first study, [79] assessed TAC and MDA, revealing a notably greater increase in these markers when compared to normal pregnant women [17]. In the second study, Lopez-Tinoco et al. [72].

Lope-Tinoco et al. 2013 measured MDA, GSH, GST, and SOD, highlighting significant increases in these markers in cases compared to the control group [72].

In the third study, [63] made a comprehensive evaluation encompassed 8-isoPGF2alpha, AOPP, PCO, GPx-3, and PON1. The findings underscored substantial elevations in these markers in GDM patients relative to normal pregnant women [72]. In the fourth study, [71] scrutinized 8-Isoprostane, 8-epiPGF2alpha, SOD, protein carbonyl, and GPx. The results indicated significant increases in all of these markers in cases compared to the control group, except for GPx, which exhibited no significant impact within the study [74].

In addition, Bartakova et al. 2015 investigated Nε-(carboxymethyl)lysine (CML) in GDM. They found significantly elevated protein- and BMI-normalized CML levels during the 24th to 30th week of gestation in women with GDM compared to healthy pregnant controls. These differences were notable even after adjusting for BMI. CML levels also correlated with 1-h and 2-h post-load glycaemia during oral glucose tolerance testing (oGTT) in GDM patients [86]. Moreover, Li and Yang (2018) investigated advanced glycation end products (AGEs) in GDM. They found significantly higher levels of AGEs in maternal plasma during both early and late stages of pregnancy in GDM compared to healthy pregnant controls [87].

#### OS markers in miscarriages

Two studies were included in our analysis of miscarriages, where various OS markers were assessed. In the first study [45], markers such as 8-OHdG, 8-NO2-Gua, HNE-MA, and MDA were examined. The results revealed significant increases in these markers when compared to the normal control group [88].

In the second study, [50] measured TOS, Prolidase, LOOH, TAC, and -SH. The findings showed a significant increase in prolidase, LOOH and TOS, along with a decrease in TAS and -SH in the case group when contrasted with the control group. Interestingly, the control group displayed lower values of Prolidase, LOOH and TOS but higher values of TAS and -SH in comparison to the case group [63].

## Discussion

In this review, we aimed to address gaps and inconsistencies in the existing literature regarding OS markers in pregnancy. The review encompassed data from a substantial number of studies, and we identified 45 of them as having a low risk of bias, which were included in the analysis. These selected studies delved deeply into OS markers. The comprehensive findings of these studies highlighted the significance of these markers in both normal and pathological pregnancy conditions. All the included studies are summarized in Table 3.

**Table 3:** Summary of all the studies included in this systematic review = 45

	Author	Study area	Study design	Sample size	Sample type	Markers assessed
*1*	*Zelanzniewick *et al*. 2015* [50]	Poland	Cross-section	34	Blood	8-iso-PGF2α and 8-OHdG
*2*	*Basu et al. 2015 * [61]	US	Cross-section	201	Placenta	MDA and TAC
*3*	*Rejc et al. 2017 * [75]	Slovenia	Cross-section	146	Urine and Amniotic fluid	8-IP and 8-OHdG
*4*	*Restini *et al*. 2018* [66]	Spain	Cross-section	253	Urine	TAC and SOD
*5*	*Roger *et al*. 2007* [71]	Hong Kong	Cohort	408	Urine	8-isoPGF2α and 2,3 8-isoPGF2α
*6*	*Draganovic et al.2016 * [62]	Bosnia	Cross-section	200	Blood	TBARS
*7*	*Vakilan *et al*. 2009* [76]	Iran	Cross-section	120	Blood	TBARS and total thiol molecule
*8*	*Jjain and Wise 1995* [77]	US	Cross-section	60	Serum	MDA
*9*	*Morris et al.1998 * [18]	UK	Cohort	45	Plasma	8-isoPGF2α and MDA
*10*	*Mannaerts et al. 2018 * [11]	Belgium	Cross-section	97	Plasma	SOD
*11*	*Bernardi et al. 2008 * [13]	Brazil	Case control	70	Serum	TBARS
*12*	*Kurlak et al. 2023 * [16]	UK	Cohort	140	Plasma	SOD, CAT and GPx
*13*	*Bogavac *et al*. 2016* [53]	Serbia	Case control	107	Blood	SOD, GSH-Px1, TAS
*14*	*Ahmed *et al*. 2019* [69]	US	Case control	114	Blood	SOD,GSH,GSSG, and CAT
*15*	*Ferguson et al. 2015 * [78]	US	Cohort	452	Blood and Urine	8-isoprostane
*16*	*Sikkema et al. 2001 * [8]	Netherland	Cross-section	23	Placental tissue	SOD
*17*	*Shigemitsu et al. 2016 * [79]	Japan	Cross-section	58	Placental tissue	MDA and TBARS
*18*	*Godhamgaonkar and Joshi 2023 * [80]	India	Cross-section	120	Plasma	MDA
*19*	*Toy et al. 2009 * [70]	Turkey	Case control	32	Serum	Prolidase,TSO,TAS,OSI and T-SH
*20*	*Mert et al. 2012 * [20]	Turkey	Case control	81	Serum	TOS and TAC
*21*	*Kressig et al. 2008 * [81]	Switzerland	Case control	58	Blood	MDA
*22*	*Biberoglu et al. 2016 * [68]	Japan	Cross-section	40	Myometrial tissue and Serum	MDA, CAT, XO and SOD
*23*	*Yoshida et al. 2018 * [64]	Japan	Case control	70	Serum and placenta	8-OHdG and dROM
*24*	*Longini et al. 2005 * [82]	Italy	Case control	114	Amniotic fluid	F2-isoprostane
*25*	*Kamath et al. 2006 * [26]	India	Cross-section	28	Blood	MDA
*26*	*Zhang *et al*. 2023* [52]	China	Cohort	206	Urine and Plasma	8-OHdG
*27*	*Biri et al. 2007 * [21]	Turkey	Case control	25	Umbilical cord plasma	MDA and XO
*28*	*Ilhan et al. 2015 * [39]	Turkey	Case control	72	Serum	8-isoprostane, TOS and TAS
*29*	*Musilova et al. 2016 * [35]	Slovakia	Cross-section	165	Umbilical cord blood	TAC and TBARS
*20*	*Longini et al, 2007 * [42]	Italy	Case control	113	Amniotic fluid	F2-isoprostane
*31*	*Ryu et at. 2017* [38]	South Korea	Cohort	72	Serum	MDA
*32*	*Ozler et al. 2016 * [43]	Turkey	Cohort	58	Amniotic fluid	TOS and TAC
*33*	*Cindrova davies *et al*. 2018* [83]	Canada	Case control	39	Placental blood	8-OHdG and H2O2
*34*	*Eick et al. 2020 * [84]	Puerta Rico	Cohort	469	Urine	8-iso-PGF2α
*35*	*Kurlak et al. 2014 * [6]	UK	Case control	305	Serum	TBARS
*36*	*Venkatesh *et al*. 2016* [85]	Israel	Cohort	366	Urine	8-OHdG and 8-isoprostane
*37*	*Hamzaoglu *et al*. 2023* [48]	Turkey	Case control	93	Serum	SOD, TOS, OSI and TAS
*38*	*Toescu et al.2004 * [17]	UK	Cohort	60	Serum	TAC and MDA
*39*	*Lopez-Tinoco *et al*. 2013* [72]	Spain	Case control	78	Serum and Urine	MDA, GSH, GST and SOD
*40*	*Li et al. 2016 * [65]	China	Case control	52	Plasma	8-isoPGF2α,AOPP,PCO1,GPx-3&PON1
*41*	*Coughlan et al. 2004 * [74]	Australia	Cohort	49	Placenta	8-isprostane,8-epiPGF2α,SOD & GPX
*42*	Bartakova et al. 2015 [86]	Czech	Case control	307	Blood	(AGEs))—Nε- (CML)
*43*	*Lı and Yang 2019* [87]	China	Case control	180	Blood	Advanced glycation end products (AGEs)
*44*	*Lin et al. 2023 * [88]	Taiwan	Cross-section	514	Urine	8-OHdG,8-isoPGF2α,8NO2Gua&MDA
*45*	*Toy et al. 2010 * [63]	Turkey	Cross-section	90	Serum	TOS,LOOH,Prolidase and TAS

This comprehensive review systematically explores OS markers in pregnancy, shedding light on their significance in both normal physiological conditions and various pathological states. In normal pregnancies, OS markers such as MDA, 8-OHdG and TBARS exhibit elevated levels, indicating an essential increase in oxidative load to support physiological processes [16, 50, 61, 63–66].

The review extends its focus to pathological pregnancy conditions, such as PE, IUGR, PROM, PBTs, GDM, and miscarriages. In these conditions, OS markers were significantly increased compared to normal pregnancies, indicating a potential contribution to the development and progression of these complication:

### Pre-eclampsia

In PE, studies consistently demonstrate significant elevations in oxidative stress markers such as MDA, 8-isoPG F2α, and SOD compared to normal pregnancies. These findings suggest a potential role of OS in the pathogenesis of PE and highlight the need for further investigation into specific markers [11, 13, 16, 18, 67, 68, 6, 69].

### Intra uterine growth restriction

Research on IUGR reveals significant increases in markers such as MDA, TOS, and TAC. These findings underscore the pervasive influence of OS in IUGR and emphasize the importance of comprehensive marker assessment across different sample types [20, 21, 26, 52, 70–74].

### Premature rupture of membrane and preterm births

Sıgnıfıcant levels of markers such as isoprostane, TBARS, and TOS have Increased compared to normal pregnancies. These findings suggest a potential association between OS and the onset of preterm labor and underscore the need for further investigation into specific markers [6, 38, 39, 42, 43, 48, 50, 75].

### Gestational diabetes mellitus

In the context of GDM, research demonstrates significant increases in markers such as MDA, TAC, 8-isoPGF2alpha and advanced glycation end products (AGEs) compared to normal pregnancies. These findings suggest a potential role of oxidative stress in the pathogenesis of GDM and highlight the need for further exploration of specific markers [66, 71, 76, 86, 87].

### Miscarriages

Studies on miscarriages reveal significant increases in oxidative stress markers such as 8-OHdG, MDA, and TOS. These findings suggest a potential association between OS and pregnancy loss, highlighting the need for further investigation into specific markers [78, 79].

Many pregnancy-related diseases characterized by OS remain etiologically unidentified. Notably, these conditions exhibit a significant elevation in oxidative stress levels. Consequently, a prevailing hypothesis among obstetricians and gynecologists suggests that OS may play a crucial role in the pathogenesis of these diseases. Therefore, the study of OS markers becomes paramount in understanding the underlying mechanisms.

For future researchers, a deeper exploration is warranted to identify the specific OS markers associated with these complications. Furthermore, unraveling the root causes that trigger heightened OS in these conditions is imperative. Such investigations will provide invaluable insights into the etiology and potential therapeutic interventions for these pregnancy-related diseases.

These studies, while valuable, exhibit certain limitations. One significant constraint lies in the absence of a comprehensive assessment of all potential OS markers that may fluctuate during pregnancy. Each study tends to focus on specific markers, and their selection is not standardized or in a specific order. For example, some studies only assessed MDA, TBARS or 8-OHdG while many other markers could provide a more holistic view of OS during pregnancy. This lack of consistency and comprehensiveness in marker evaluation poses a considerable challenge when attempting to perform a meta-analysis within the scope of this review (Table 3). The absence of a holistic approach to oxidative stress assessment limits the ability to draw comprehensive and interconnected conclusions.

To address this limitation and improve the understanding of OS in pregnancy, future studies should consider assessing a broader range of OS in a standardized manner. Moreover, there is a need to explore various sample types in future studies. Assessing the markers in different sample types such as blood, placenta, amniotic fluid, myometrial tissue, Urine, Plasma, serum, and umbilical cord blood in a single study would provide a more comprehensive understanding of OS in different maternal and fetal compartments. Despite these limitations, the findings underscore the pivotal role of OS in pregnancy-related diseases, prompting further investigations into specific markers and their implications. The review provides valuable insights into the intricate relationship between OS and pregnancy, paving the way for future studies to unravel underlying mechanisms and explore potential therapeutic interventions. However, it’s essential to approach the interpretation of these findings with caution due to variations in study designs, populations, and methodologies, emphasizing the need for continued research to deepen our understanding of OS in pregnancy and its implications for maternal and fetal health.

## Conclusion

In conclusion, this systematic review has provided a comprehensive analysis of OS markers in pregnancy, including both normal and pathological pregnancy conditions. The review identified 45 studies with a low risk of bias that investigated a wide range of OS markers. The findings underscore the significance of these markers in pregnancy and pregnancy-related complications.

This review contributes to the existing literature by filling gaps and inconsistencies in the understanding of oxidative stress in pregnancy. It serves as a valuable resource for researchers and healthcare professionals interested in this field, paving the way for further research to explore the mechanisms and implications of OS during pregnancy.

The complex interplay between OS and pregnancy conditions remains an area of ongoing investigation, and this systematic review offers a solid foundation for future research endeavors in this vital area of women’s health. It is clear that OS markers hold the key to unraveling the mysteries of various pregnancy-related complications, and further exploration is necessary to fully comprehend their role in maternal and fetal health.

### Supplementary Information

Supplementary Material 1.

## Data Availability

No datasets were generated or analysed during the current study.
