# Supplementary material for: Oxidative stress biomarkers in pregnancy: a systematic review
Source: Reprod Biol Endocrinol. 2024 Aug 2;22:93. doi: 10.1186/s12958-024-01259-x (PMC11295331; doi:10.1186/s12958-024-01259-x)
Supplement: Supplementary file 1 — Supplementary Material 1. [file 12958_2024_1259_MOESM1_ESM.docx]

1. Borowiecka M, Wojsiat J, Polac I, Radwan M, Radwan P, Zbikowska HM. Oxidative stress markers in follicular fluid of women undergoing in vitro fertilization and embryo transfer. Syst Biol Reprod Med. 2012 Dec;58(6):301-5. doi: 10.3109/19396368.2012.701367. Epub 2012 Sep 5. PMID: 22950633.

Detailed Rationale for Exclusion

Relevance to Gynecological Conditions: The study focuses on IVF outcomes rather than pregnancy conditions such as preeclampsia (PE), intrauterine growth restriction (IUGR), preterm birth, etc. This makes it less relevant for a systematic review specifically aimed ın assessıng oxidative stress markers in pregnancy and pregnancy-related complications.

Potential Confounders Not Addressed: The study does not adequately address potential confounders such as age and BMI, which are significantly different between groups and could influence the results.

Specificity to IVF: The findings may not be generalizable to natural pregnancies due to the unique physiological and hormonal conditions associated with IVF.

Conclusion

Given the identified biases, especially the lack of generalizability to natural pregnancy conditions and inadequate control of confounders, this study is excluded from our systematic review. This decision ensures that the included studies are directly relevant to understanding oxidative stress in both physiological and pathological pregnancy conditions without the confounding factors specific to IVF treatment.

RISK ASSESSMENT % = 60

2. Khan N, Lambert-Messerlian G, Monteiro JF, Hodosy J, Tóthová Ľ, Celec P, Eklund E, Curran P, Bourjeily G. Oxidative and carbonyl stress in pregnant women with obstructive sleep apnea. Sleep Breath. 2018 Mar;22(1):233-240. doi: 10.1007/s11325-017-1475-8. Epub 2017 Feb 24. PMID: 28236270; PMCID: PMC5568979.

Detailed Rationale for Exclusion

Relevance to Gynecological Conditions: The study focuses specifically on OSA, which is not a primary gynecological condition but rather a comorbid condition that may affect pregnancy outcomes. It does not alıgned wıth our prımary focus.

Contradictory Findings: The study's findings of higher antioxidant capacity and lower oxidative stress markers in women with OSA contradict the initial hypothesis and the general understanding of oxidative stress in pathological pregnancy conditions. This discrepancy may introduce complexity and potential confusion in our systematic review's narrative.

Inadequate Control for Confounders: Despite adjusting for some confounders, other important factors like diet, lifestyle, and severity of OSA are not controlled for, which can significantly influence oxidative stress levels.

Conclusion

Given the identified biases, particularly the relevance to gynecological conditions and the potential for confounding and measurement biases, this study is excluded from OUR systematic review. This ensures that the included studies directly address oxidative stress in pregnancy-related complications without the confounding factors specific to OSA.

RISK ASSESSMENT % = 35

1. Gümüş P, Emingil G, Öztürk VÖ, Belibasakis GN, Bostanci N. Oxidative stress markers in saliva and periodontal disease status: modulation during pregnancy and postpartum. BMC Infect Dis. 2015 Jul 8;15:261. doi: 10.1186/s12879-015-1003-z. PMID: 26152310; PMCID: PMC4495776.

Detailed Rationale for Exclusion

Relevance to Gynecological Conditions: The primary focus of the study is on periodontal disease and its impact on oxidative stress levels in pregnant and postpartum women. While periodontal health may influence systemic health and pregnancy outcomes, it is not directly related to gynecological conditions which are the main focus of our systematic review.

Measurement and Confounding Issues: The study's reliance on saliva as the sole source for measuring oxidative stress markers, without controlling for various confounders, raises concerns about the accuracy and applicability of the findings to the broader context of oxidative stress in pregnancy and pregnancy-related complications.

Given these biases, particularly the relevance to the main focus of the systematic review and potential confounding and measurement biases, this study is excluded from the systematic review.

RISK ASSESSMENT % =40

4.Hu YY, Liu JC, Xing AY. Oxidative stress markers in intrahepatic cholestasis of pregnancy: a prospective controlled study. Eur Rev Med Pharmacol Sci. 2015 Sep;19(17):3181-6. PMID: 26400520.

Relevance to Gynecological Conditions:

The study focuses on intrahepatic cholestasis of pregnancy (ICP), a condition that can impact pregnancy outcomes. However, our systematic review aims to assess oxidative stress markers in a broader range of gynecological conditions The narrow focus on ICP and its specific oxidative stress markers dıd not fully align with the broader scope of the review.

Measurement and Confounding Issues:

The study measures oxidative stress markers in maternal plasma, which is appropriate for assessing systemic oxidative stress. However, potential confounders such as variations in diet, medication use, and other health conditions that might influence oxidative stress levels were not controlled for in the study. As they mentıon ın the study patients were admitted into the study regardless of their previous treatment. This could impact the accuracy and generalizability of the findings.

Given these biases, particularly the limited relevance to the broader scope of the systematic review, potential confounding, and selection biases, this study is excluded from the systematic review.

RISK ASSESSMENT % =50

5. Asemi Z, Samimi M, Tabassi Z, Sabihi SS, Esmaillzadeh A. A randomized controlled clinical trial investigating the effect of DASH diet on insulin resistance, inflammation, and oxidative stress in gestational diabetes. Nutrition. 2013 Apr;29(4):619-24. doi: 10.1016/j.nut.2012.11.020. PMID: 23466048.

Relevance to Gynecological Conditions:

The study focuses on the effects of the DASH diet on insulin resistance, inflammation, and oxidative stress in pregnant women with gestational diabetes mellitus (GDM). While GDM is a relevant gynecological condition associated with oxidative stress, the primary intervention in this study is dietary modification rather than a direct investigation of oxidative stress markers in the context of pregnancy or pregnancy complications like preeclampsia (PE), intrauterine growth restriction (IUGR), preterm birth, and others.

Measurement and Confounding Issues:

The study measures a range of biomarkers, including fasting plasma glucose (FPG), serum insulin, homeostasis model of assessment-insulin resistance (HOMA-IR), plasma total antioxidant capacity (TAC), and total glutathione (GSH). However, the focus on dietary intervention introduces potential confounding factors such as differences in baseline nutritional status, and other lifestyle factors that could influence the outcomes. These confounders make it challenging to isolate the effect of oxidative stress from the dietary intervention itself.

Selection and Information Bias:

The randomized controlled trial design helps mitigate selection bias, but the exclusıon of the ındıvıduals with premature preterm rupture of membrane, placenta abruption, preeclampsia, and those who needed to commence insulin therapy or were on insulin therapy, as well as those with a recommendation for complete bed rest and that may limit the generalizability of the findings. Additionally, the study. Information bias other unmeasured lifestyle factors that were not controlled for in the analysis.

Given these biases:

Relevance to the Main Focus: The primary focus on dietary intervention rather than direct measurement of oxidative stress markers in a broad range of pregnancy-related complications.

Potential Confounding: The introduction of various confounding factors related to dietary adherence and baseline nutritional status.

Selection : Exclusıon of the ındıvıduals with premature preterm rupture of membrane, placenta abruption, preeclampsia, and those who needed to commence insulin therapy or were on insulin therapy, as well as those with a recommendation for complete bed rest and that may limit the generalizability of the findings.

RISK ASSESSMENT %=60

6. Özyer S, Ozel S, Karabulut E, Kahyaoglu S, Neselioglu S, Erel O, Engin-Ustun Y. Oxidative-Antioxidative Markers in Pregnant Women with Fetal Neural Tube Defects. Fetal Pediatr Pathol. 2021 Apr;40(2):93-102. doi: 10.1080/15513815.2019.1686783. Epub 2019 Nov 25. PMID: 31762366.

Relevance to Gynecological Conditions:

This study focuses specifically on comparing markers of oxidative stress in pregnant women with and without fetal neural tube defects (NTDs). While NTDs are indeed relevant to pregnancy and fetal health, the direct measurement of oxidative stress markers in this specific context dıd not align perfectly with the broader scope of our systematic review. The focus on NTDs, although important, limit the generalizability of the findings to other gynecological conditions.

Measurement and Confounding Issues:

The study employs a robust methodology by including matched control mothers without NTD-affected fetuses, which helps control for potential confounders related to age, gestational age, and body mass index. However, there could still be confounding factors that are not accounted for in the analysis e.g underlying health conditions that may influence oxidative stress markers independently of NTDs. Additionally, while the study measures several parameters related to thiol-disulfide homeostasis and ischemia-modified albumin (IMA), it does not provide information about other potential markers of oxidative stress that could be relevant to our systematic review

Selection and Information Bias:

The inclusion of matched control mothers is a strength in minimizing selection bias. However, the sample size of 30 affected mothers and 31 controls, while adequate for a comparative study, there is a lack of detailed information in the about the selection criteria for participants and any potential biases that might impact the representativeness of the sample.

Given these considerations:

Relevance to the Main Focus: While the study addresses oxidative stress markers in pregnancy, its specific focus on NTDs may not fully align with the broader scope of our systematic review on various pregnancy-related complications.

Potential Confounding: Although efforts are made to match control mothers, there may still be unaccounted confounding factors that could influence oxidative stress marker levels independently of NTDs.

Selection and Information Bias: There is a lack of detailed information about participant selection criteria and potential biases.

RISK ASSESSMENT %=55

7. Cakina S, Pek E, Ozkavak O, Kocyigit D, Beyazıt F. The role of paraoxonase and myeloperoxidase as oxidative stress markers in pregnant women with hypothyroidism. Gynecol Endocrinol. 2022 Oct;38(10):840-843. doi: 10.1080/09513590.2022.2114449. Epub 2022 Aug 23. PMID: 35999709.

Relevance to Gynecological Conditions: We excluded the study on hypothyroidism during pregnancy because while it can impact maternal and fetal health, it does not directly align with our specific focus on oxidative stress markers in gynecological complications. Our study primarily targets conditions like preeclampsia (PE), intrauterine growth restriction (IUGR), preterm birth, and others. Since hypothyroidism is not one of these targeted gynecological diseases, it may not contribute directly to our research objectives.

Measurement and Confounding Issues: Although the study investigates paraoxonase 1 (PON 1) and myeloperoxidase (MPO) levels in hypothyroid pregnant patients, it does not delve deeply into oxidative stress markers within the context of gynecological complications. The focus on PON 1 and MPO levels, while relevant to hypothyroidism, does not directly address the broader spectrum of oxidative stress markers related to our targeted gynecological conditions.

Selection and Information Bias: Our exclusion was also influenced by the study's inclusion criteria, which encompassed 45 hypothyroid pregnant patients and 45 healthy control pregnant individuals based on age compatibility. However, without additional information on other potential confounders or selection biases related to oxidative stress in gynecological complications, it's challenging for us to ascertain the study's relevance to our systematic review.

Relevance to the Main Focus: Given the specific focus of our systematic review on oxidative stress markers in a range of gynecological complications, the study on hypothyroidism during pregnancy may not provide direct insights or data that align with our research questions and objectives.

Therefore, based on these considerations, we excluded the study on hypothyroidism during pregnancy to ensure that the included studies directly address oxidative stress markers in the context of our targeted gynecological complications without introducing potential confounding factors or diverging from our main research focus.

RISK ASSESSMENT %=40
